# Supplementary material for: BmSuc1 Affects Silk Properties by Acting on Sericin1 in Bombyx mori
Source: Int J Mol Sci. 2022 Aug 31;23(17):9891. doi: 10.3390/ijms23179891 (PMC9456260; doi:10.3390/ijms23179891)
Supplement: Supplementary file 1 [file ijms-23-09891-s001.zip › supplementary materials.pdf]

BmSuc1 Affects Silk Properties by Acting on Sericin1 in *Bombyx mori*

Liangli Yang, Yue Zhao, Quan Gan, Dan Liang, Rui Shu, Song Jiang, Ruiping Xie, Yan Meng

SUPPLEMENTARY DATA

Table S1. Oligonucleotide primers used in PCR.

Table S2. sgRNA sequence.

Figure S1. Schematic diagram of the transgenic plasmid and an experimental diagram for generation of the homozygous mutant lines.

Figure S2. PCR detection results of the hybrid progeny gDNA.

Figure S3. Prokaryotic expression of ΔBmSUC1 and BmSUC1.

Figure S4. Changes of sucrose hydrolase activity in larval midgut after knockout BmSuc1.

Table S1. Oligonucleotide primers used in PCR. Boldface nucleotides indicates protection base. Underline indicate the restriction sites. Double underscores indicate Flag label.

Name of primer	Primer sequence (5' to 3')
RT-PCR-*BmSuc1*-F	CAGCTGTACATATGTTCGCCTGGAGCACAC
RT-PCR-*BmSuc1*-R	CGGCTCGAGAGCGGGTACACTTCTTCTCAATC
qRT-PCR-*BmSuc1*-F	ACTTACTTGATTGGCTGGTT
qRT-PCR-*BmSuc1*-R	CTTCCGTTATGGACGCTAT
RT-PCR-*BmActin3*-F	AACACCCCGTCCTGCTCACTG
RT-PCR-*BmActin3*-R	GGGCGAGACGTGTGATTTCCT
qRT-PCR-*Bmrp49*-F	CCCAACATTGGTTACGGTTC
qRT-PCR-*Bmrp49*-R	GCTCTTTCCACGATCAGCTT
sgRNA1-F	GTCGACGGCTGGATGAAT
sgRNA1-R	GTCGACAAAAAAAGCACCG
sgRNA2-F	GCTAGCGCTTCTACGGACAA
sgRNA2-R	GCTAGCAAAAAAAGCACCAG
sgYZ F	AGGTTATGTAGTACACATTGTTGTA
sgYZ R	ATTATCTTTTACGTGACTTTTAAGA
BmSuc1 ORF-F	ATGTTCGCCTGGAGCACACC
BmSuc1 ORF-R	TTAAGCGGGTACACTTCTTC
△*BmSuc1*- ORF-F	**CCC**AAGCTTATGGATTACAAGGATGACGACGATAAGATGCTCCGCCAGCAAAAT
△*BmSuc1*- ORF-R	**ATA**CTCGAGCACGCTTAAGCGGGTA

Table S2. sgRNA sequence. Bold is the PAM site, bold and italics indicate the restriction site *Sal* I or *Nhe* I, the underline is the target sequence of BmSuc1, and the rest are transRNA

Name	target sequence	sgRNA sequence (5' to 3')
sgRNA1	5'-GGCTGGATGAATGACCCTAA**CGG**-3'<br>3'-CCGACCTACTTACTGGGATTGCC-5'	***GTCGAC***GGCTGGATGAATGACCCTAAGTTTTAGAGCTAGAAATAGCAAGTTAAAATAAGGCTAGTCCGTTATCAACTTGAAAAAGTGGCACCGAGTCGGTGCTTTTTTT***GTCGAC***
sgRNA2	5'-CCGCAAACCTTGTCCGTAGAAGC-3'<br>3'-**GGC**GTTTGGAACAGGCATCTTCG-5'	***GCTAGC***GCTTCTACGGACAAGGTTTGGTTTTAGAGCTAGAAATAGCAAGTTAAAATAAGGCTAGTCCGTTATCAACTTGAAAAAGTGGCACCGAGTCGGTGCTTTTTTT***GCTAGC***

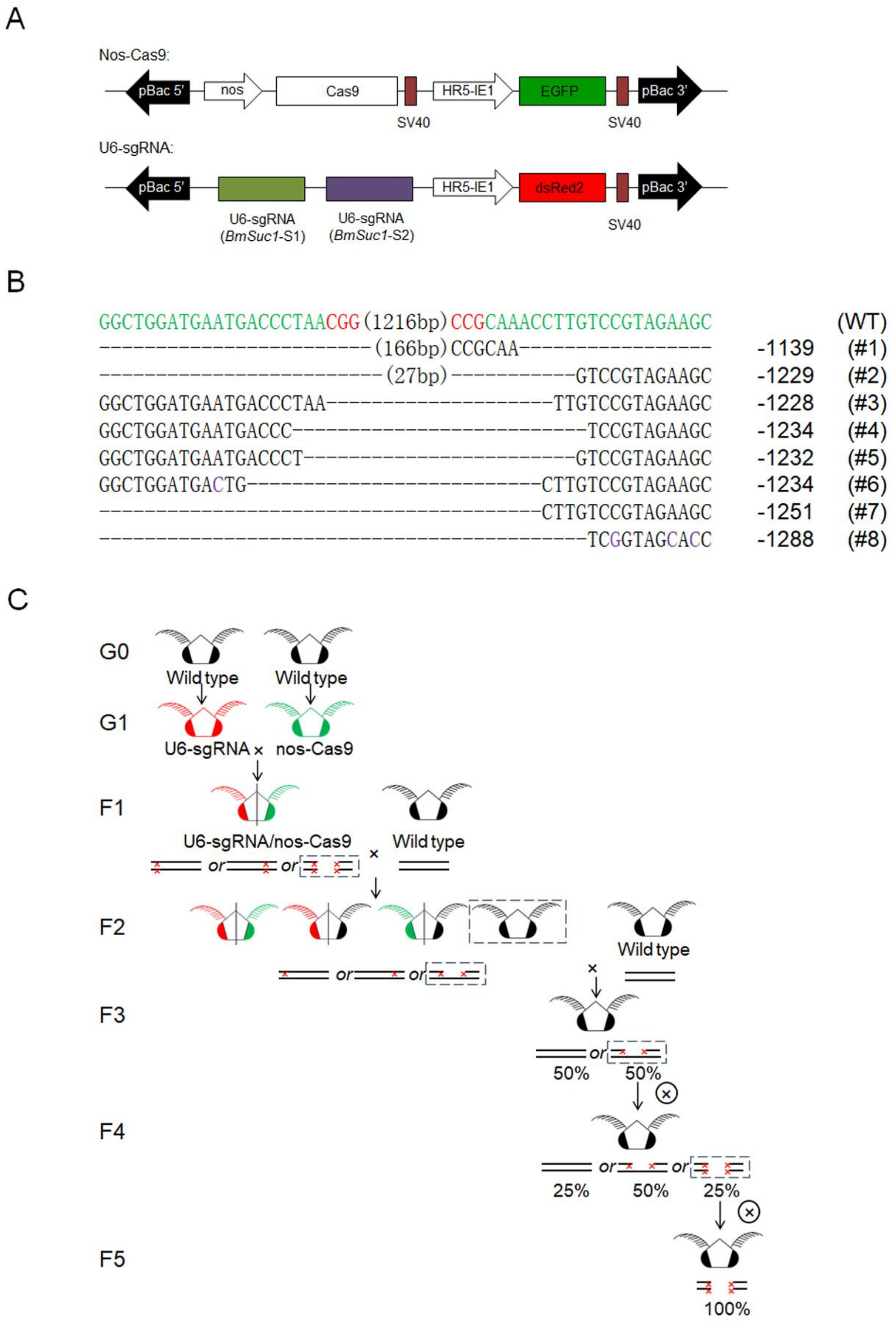

Figure S1. Schematic diagram of the transgenic plasmid and an experimental diagram for generation of the homozygous mutant lines. (A) Structural sketches of two transgenic plasmids. (B) Various types of non-deletion or deletion mutations in F1 generation *BmSuc1* mutants. The sgRNA-targeting sequence is in green, and the PAM

sequence is in red. The number of 1216 bp in brackets refers to the 1216 bp interspace fragment between the two target sites. (C) Hybridization strategies to produce *BmSuc1* homozygous mutants. (1) The transgenic plasmids U6-sgRNA or Nos-Cas9 were injected into the pre blastoderm silkworm embryos to produce two transgenic silkworm lines (G1). (2) Subsequently, founder animals (F1) who expressed both *BmSuc1* sgRNAs and Cas9 were produced by the hybridization of the two transgenic lines. (3) The F2 progeny were obtained by backcrossing the F1 somatic mutant with WT, then the F2 progeny lacking fluorescence with complete deletion events were backcrossed with WT moths again to obtain F3 progeny that were 50% heterozygous mutants and 50% WT. (4) The F4 progeny that were 25% WT, 50% heterozygous mutants, and 25% homozygous mutants were obtained by sib-mating of F3 heterozygous moths. (5) Finally, the F5 progeny that were 100% homozygous were obtained by sib-mating the F4 homozygous moths. Two *BmSuc1* allele mutant lines were established.

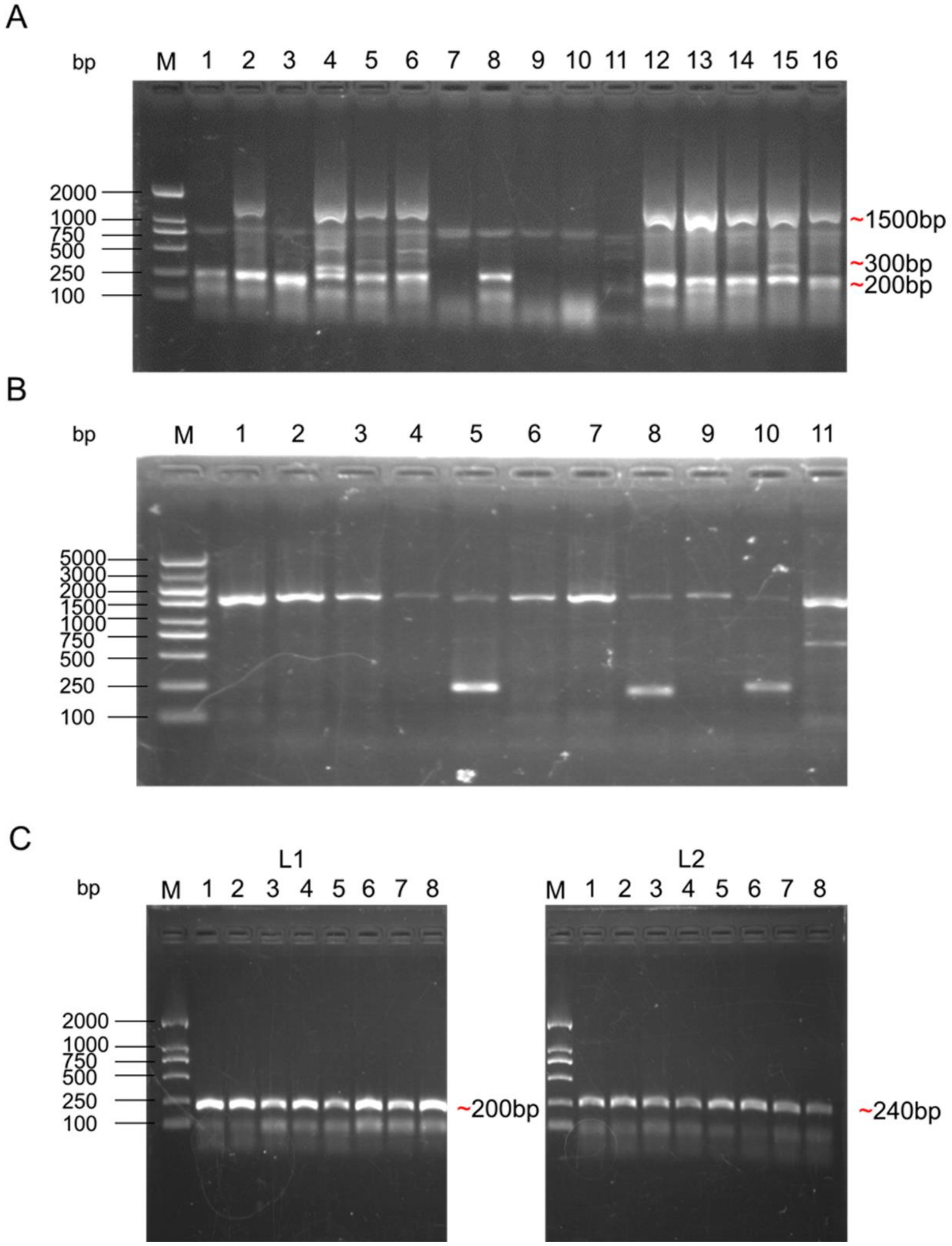

Figure S2. PCR detection results of the hybrid progeny gDNA. (A) Results of PCR analysis of the F1 generation *BmSuc1* mutation adults. Lanes 1–16 were all amplified fragments from the genome of the mutant *Bombyx mori*. (B) Results of PCR analysis of the F2 progeny that lacked fluorescence. (C) Mutation detection results of two obtained *BmSuc1* homozygous mutant strains L1 and L2. Lanes 1–8 were PCR amplification results of 8 randomly selected silkworm genomes from two strains of *Bombyx mori* in the F5 generation.

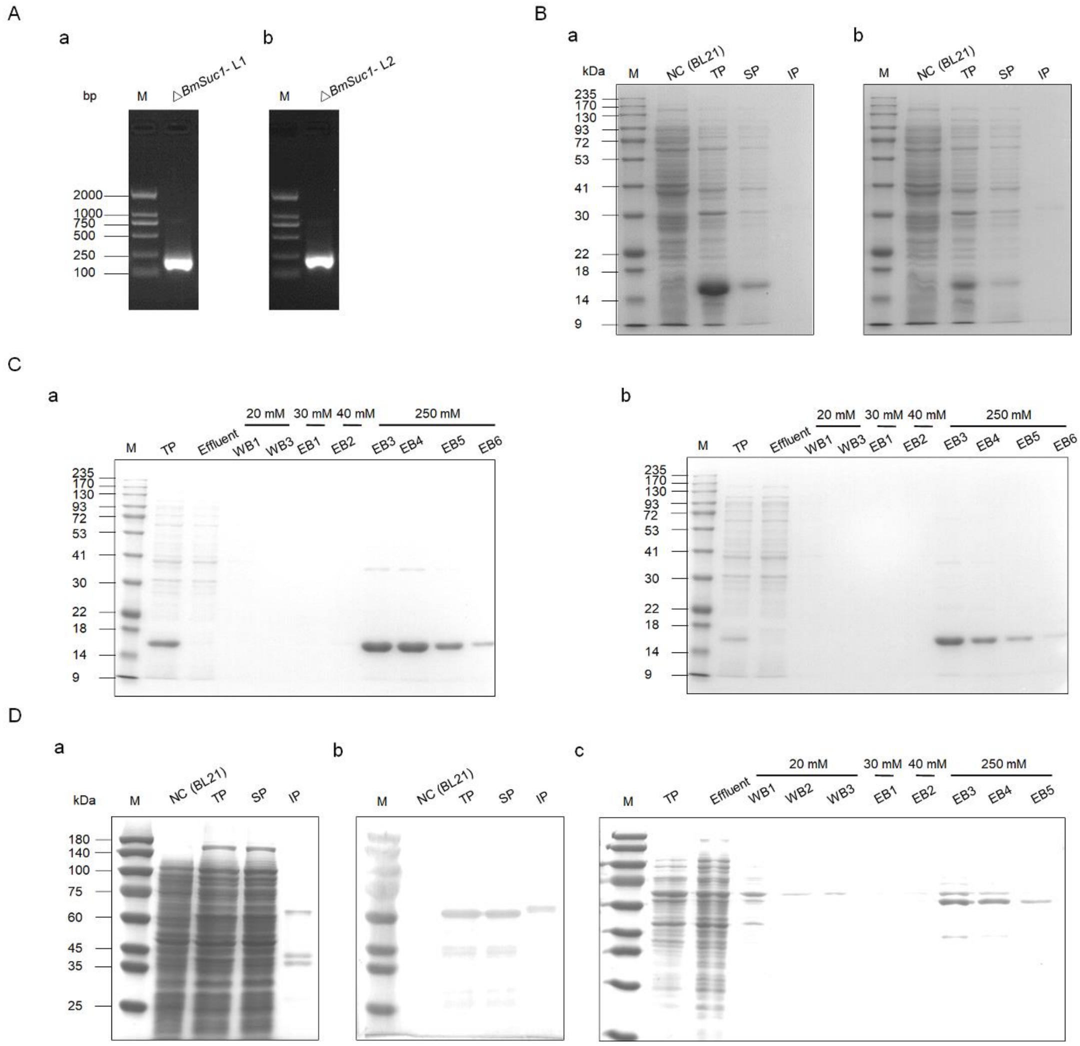

Figure S3. Prokaryotic expression of ΔBmSUC1 and BmSUC1. (A) The ORF of truncated *BmSuc1* in mutant L1 and L2 were amplified by PCR. (B) The expression of ΔBmSUC1 induced by isopropyl β-D-thiogalactoside (IPTG) was detected as soluble protein by SDS-PAGE. (C) Purification of soluble protein ΔBmSUC1. a, ΔBmSUC1-L1; b, ΔBmSUC1-L2. (D) The expression of BmSUC1 induced by IPTG was detected as soluble protein by (a) SDS-PAGE and (b) western blot using anti-BmSUC1 antibody, and (c) purification of soluble protein BmSUC1 (TP, total protein; SP, soluble protein; IP, inclusion body protein; WB, washing buffer; EB, elution buffer.).

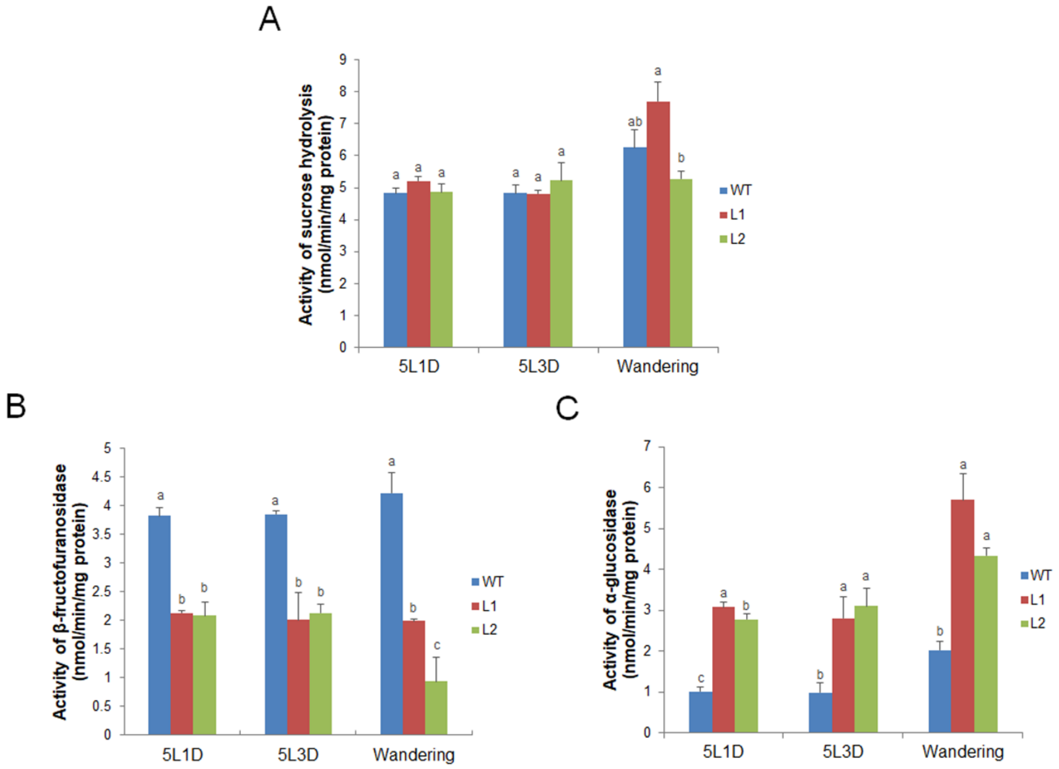

Figure S4. Changes of sucrose hydrolase activity in larval midgut after knockout *BmSuc1*. (A) Sucrose hydrolase activity (-DNJ), (B) β-fructofuranosidase activity (+DNJ), and (C) α -glucosidase activity of total midgut proteins at the 5L1D, 5L3D, and wandering stages.
